# Artificial intelligence and its influence on student engagement in higher education

**DOI:** 10.3389/fpsyg.2026.1949332

**Published:** 2026-10-15

**Authors:** Yongsheng Chen, Jing Dong, Guojuan Ji

**Affiliations:** 1 Yangzhou University, Yangzhou, China; 2 Tongda College of Nanjing University of Posts and Telecommunications, Yangzhou, China

**Keywords:** agentic engagement, AI conversational agent, Chinese universities, cultural adaptation, mixed-methods design, quasi-experimental design, Socratic dialogue

## Abstract

Empirical evidence on how artificial intelligence (AI) relates to student engagement in culturally diverse higher-education settings remains limited. In Chinese university classrooms, where interaction is often teacher-led and asking questions in class can carry a social cost, AI-driven dialogic tools may offer low-pressure opportunities for active participation. This study examined the association between an AI conversational agent (EduBot) and the behavioural, cognitive, emotional and agentic dimensions of engagement among Chinese undergraduates. A mixed-methods quasi-experimental design involved 555 students in four comprehensive (multi-disciplinary, research-intensive) universities, each contributing one introductory course taught in two parallel sections. Within a 15-week semester, intervention sections (n = 278) used EduBot, a Socratic-dialogue agent embedded in the learning management system, for 12 weeks; control sections (n = 277) used teaching-assistant-moderated forums. We measured engagement at three time points with a 21-item four-dimensional engagement scale adapted from an established measure and supplemented it with system logs, 30 student interviews and instructor focus groups. Linear mixed-effects models accounting for the nesting of students within course sections, together with mixed-design ANOVAs, showed Group × Time interactions for behavioural (η^2^p = 0.043, post-test d = 0.71), cognitive (η^2^p = 0.032, d = 0.55), agentic (η^2^p = 0.054, d = 0.57) and emotional engagement (η^2^p = 0.012, d = 0.34), all *p* ≤ 0.001 after Holm adjustment. Within the intervention group, the number of EduBot sessions predicted post-test engagement after adjustment for baseline scores and covariates. Exploratory moderation analyses indicated larger agentic gains among first-year students and larger cognitive gains among students with lower prior GPA. Thematic analysis identified three hypothesised mechanisms, namely a judgment-free interaction space, scaffolded persistence and temporal flexibility. A Socratic-scaffolded AI agent was associated with higher engagement, most clearly for agentic engagement, which participants described as constrained by classroom norms. The findings support culturally attentive AI design and suggest, tentatively, an equity-enhancing potential. Because the design compared an integrated AI package with usual practice across eight sections, the effects cannot be attributed to single design components.

## 1 Introduction

The nature of the AI transformation in higher education is one of deep change due to the fast integration of artificial intelligence (AI) technologies (Pireci Sejdiu and Sejdiu, 2025). Based on predictive analytics, generative AI systems, adaptive tutoring, and intelligent chatbots, these technologies are changing the essence of the learning process and student interaction (Shahzad et al., 2024; Chakraborty, 2026). Student engagement—an investment in learning behaviourally, cognitively, and emotionally—is widely known as a defining factor of academic achievement (Xu et al., 2023). However, conventional pedagogical designs struggle to maintain this engagement on a large scale (Biku et al., 2018). AI-based learning devices may offer a solution, raising the critical question of how AI can be used to increase student engagement (Zhou and Peng, 2025). The use of AI applications in higher education has grown exponentially, focusing on personalised learning and tutoring systems (Vieriu and Petrea, 2025; Zhou and Peng, 2025). Systematic reviews reveal that AI tools provide beneficial additions to engagement when implemented carefully (Debets et al., 2025; Tbaishat and Elfadel, 2025). Generative AI (GenAI) applications like ChatGPT have recorded increased student confidence, motivation, and engagement across disciplinary settings (Ashraf et al., 2025; Kumar, 2021; Nasr et al., 2025), yielding significant positive effects on cognitive, behavioural, and emotional dimensions (Lin and Chen, 2024; Zhu and Yang, 2026). AI now possesses the capacity to provide personalised scaffolds and fine-tune results at scale (Rodríguez-Ortiz et al., 2025; Yan et al., 2025). Despite this accumulation of evidence, AI does not have a uniform effect on engagement rates (Long et al., 2026). AI tools provide the highest engagement when integrated into interactive pedagogies (Wang and Li, 2026), but engagement may be reduced when implementation is not considered pedagogically (Akgun and Greenhow, 2022). Frameworks like PMAISE attempt to chart this correspondence (Bulut, 2026). Furthermore, empirical findings remain patchy, often limited to small case-studies (Ofem et al., 2024). Achieving AI’s transformative potential requires a holistic ecosystem rather than *ad hoc* implementation (Lan and Zhou, 2025), alongside addressing ethical, equity, and implementation challenges to prevent propagating institutional imbalances (Balalle and Pannilage, 2025).

Several studies have examined the link between AI tools and student engagement in higher education directly, and their designs help to locate the present study. In a meta-analysis of 24 randomised studies, Wu and Yu (2024) found a large average effect of AI chatbots on learning outcomes, with larger effects in higher education than in school settings and moderation by intervention duration. Deng et al. (2025) synthesised 69 experimental studies of ChatGPT and reported consistent gains in learning performance together with less consistent effects on affective outcomes. At the level of single courses, Essel et al. (2022) randomised 68 Ghanaian undergraduates to a course chatbot or instructor-led support and found higher achievement and more confident participation in the chatbot cohort; Kumar (2021) documented improved team-based learning outcomes with an educational chatbot in a design course; Lin and Chen (2024) related AI-integrated applications to college students’ creativity and academic emotions; and Zhou and Peng (2025) modelled learning engagement as a mediator between AI use in teaching and students’ creativity, with AI literacy as a moderator. Reviews by Labadze et al. (2023) and Long et al. (2026) conclude that the benefits depend on how the tool is integrated with teaching, and that most primary studies are short, single-site and rely on self-reported outcomes without separating the behavioural, cognitive, emotional and agentic facets of engagement. Few of these studies were conducted in mainland Chinese universities, and none, to our knowledge, combined repeated measurement of a four-dimensional engagement construct with log data and qualitative accounts of how students experienced the tool.

Crucially, empirical research often overlooks how culturally specific learning environments shape AI’s pedagogical potential. Classroom interaction in many Chinese universities remains teacher-led, and studies of classroom silence report that concern about face and about interrupting the teacher can restrain students’ questioning in class (Liu et al., 2026; Shi, 2026); these patterns vary across institutions, disciplines and individual classrooms and should not be treated as fixed cultural traits (Ryan and Louie, 2007). Introducing AI-driven, dialogic tools into this distinctive environment may interact in complex ways with established behaviours. Against this background, this article investigates the association between access to an AI-based conversational agent (EduBot) and the engagement of undergraduate students in China, without assuming that the technology determines its effects. This research addresses two questions: (1) How does access to a Socratic AI conversational agent relate to change in the behavioural, cognitive, emotional and agentic dimensions of student engagement over a semester, after accounting for the nesting of students within course sections? (2) How do students and instructors describe the experiences through which the tool relates to engagement in their classroom context?

## 2 Materials and methods

### 2.1 Research design and participants

This study employed a mixed-methods quasi-experimental design with a non-equivalent control group (Natto, 2026). Four research-intensive comprehensive universities in China (Zhejiang University, Nanjing University, Wuhan University and Fudan University) took part; we recruited them purposively to limit variation in instructional infrastructure and digital readiness; comprehensive universities are multi-disciplinary institutions offering programmes across the natural sciences, engineering, humanities and social sciences, as distinct from single-discipline institutions. Each university contributed one large introductory course, namely introductory psychology at Zhejiang University, introductory computer science at Nanjing University, introductory business at Wuhan University and introductory biology at Fudan University. University and course are therefore completely confounded in this design, and institutional and disciplinary influences cannot be separated. Each course was offered in Fall 2025 in two parallel sections taught by the same instructor with the same syllabus, lecture materials, assessments and teaching-assistant support; students had been allocated to sections through routine timetable enrolment before the study was announced. Within each course, one section was assigned to the EduBot condition and the other to the control condition by a coin toss performed by the course coordinator before the semester began, yielding eight class sections (four per condition). Students were thus nested within sections, and sections within universities.

Of the 612 students enrolled in the eight sections, 583 (95.3%) were 18 years or older, consented in writing and completed the baseline questionnaire. Of these, 555 (95.2%; intervention *n* = 278, control *n* = 277) completed all three questionnaire waves and passed the attention checks, and they form the complete-case analytical sample; 28 students (12 intervention, 16 control) were lost, 21 because they missed at least one wave and 7 because a questionnaire failed the quality criteria described below. Attrition did not differ by condition (χ^2^(1) = 0.56, *p* = 0.46), and students who dropped out did not differ from completers in baseline engagement (all *p* > 0.11) or GPA (*p* = 0.16). The intent-to-treat population comprises all 583 consenting students. An *a priori* power calculation in G*Power (Kang, 2021) had indicated that 555 students provide power of 0.98 to detect a between-group difference of d = 0.35 at α = 0.05 under independence. Because students were clustered in eight sections, we recalculated power for the Group × Time contrast with the cluster-adjusted approach of Hedges and Hedberg (2007), assuming a pre–post correlation of 0.55 and four sections per arm with a mean of 69 students. Power was 0.79 for d = 0.35 at an intraclass correlation (ICC) of 0.01 and 0.55 at an ICC of 0.03, and 0.98 and 0.84, respectively, for d = 0.50. The observed section-level ICCs for engagement were 0.019–0.038 (Table 1). The design was therefore adequately powered for medium-to-large effects but not for small ones once clustering is taken into account, which we note as a limitation. The Institutional Review Board of Zhejiang University approved the protocol (ZJU-IRB-2025-042), and all participants provided written informed consent.

**Table 1:** Linear mixed-effects models of Group × Time effects on engagement, with random intercepts for students and course sections (complete-case sample, *n* = 555 students, 8 sections, 1,665 observations).

Engagement dimension	Group × T2, b (SE)	Group × T3, b (SE)	95% CI for Group × T3	Holm-adjusted *p* (Group × T3)	Wald χ^2^(2), Group × Time	ICC, section	ICC, student
Behavioural engagement	0.35 (0.08)	0.67 (0.08)	[0.51, 0.84]	< 0.001	64.12	0.019	0.541
Cognitive engagement	0.24 (0.08)	0.48 (0.08)	[0.33, 0.64]	< 0.001	36.67	0.038	0.511
Emotional engagement	0.19 (0.08)	0.34 (0.08)	[0.18, 0.49]	< 0.001	17.83	0.034	0.592
Agentic engagement	0.40 (0.09)	0.55 (0.09)	[0.37, 0.72]	< 0.001	40.62	0.024	0.583

Models: engagement ~ Group + Time + Group × Time with random intercepts for students nested in course sections; restricted maximum likelihood; reference category is the control group at T1, so b is the difference in change from T1 between conditions in scale points (1–7). ICC = intraclass correlation (proportion of total variance). All unadjusted p for Group × T3 were < 0.001. In every model the variance of section-level time slopes was estimated at zero (likelihood-ratio χ^2^(2) = 0.00), so the random-intercept model was retained; adding the slopes left the estimates unchanged to two decimals.

### 2.2 Study context and AI intervention description

The four courses were taught in a blended mode with the same lecture materials in both sections of each course.

*Timeline*. The semester lasted 15 weeks. Students completed the baseline questionnaire (T1) in week 1, before EduBot was activated; EduBot was available to the intervention sections from the start of week 2 to the end of week 13, an active intervention period of 12 weeks (84 days), which is the reference period for all log-based metrics; they completed the mid-intervention questionnaire (T2) in week 7 and the post-intervention questionnaire (T3) in week 14, the week after EduBot access was withdrawn. Interviews and focus groups took place in weeks 14–15. The intervention itself therefore lasted 12 weeks within a 15-week semester.

*EduBot*. EduBot was a conversational agent built on an open-weight large language model (Qwen2-72B-Instruct) hosted on the universities’ own servers, so that no student data left the institutions. The model parameters were not modified; pedagogical behaviour was implemented through (a) retrieval-augmented generation over each course’s lecture slides, textbook chapters and problem sets, so that responses were grounded in course materials and displayed the source passage, and (b) a fixed system prompt encoding the tutoring rules (Alsafari et al., 2024). The rules were: do not give the final answer to a problem on the first request; begin by asking the student to state what they already know or have tried; respond with a guiding question or a partial hint, offering at most three successive hints before a worked explanation; ask the student to summarise the reasoning at the end of an exchange; state explicitly when a question is outside the course materials or when the answer is uncertain; and decline requests unrelated to the course. A Socratic dialogue was logged as completed when the student reached and confirmed a solution after at least two guiding turns. A typical exchange in the biology course began with a student asking why ATP yield differs between aerobic and anaerobic respiration; EduBot asked where in the cell each pathway occurs, then which step regenerates NAD+, and only after the student connected the electron transport chain to oxygen availability did it summarise the reasoning and invite the student to restate it. Multimodal clarification allowed students to upload a photograph of a diagram or handwritten working and receive a text explanation; metacognitive prompts appeared at the end of each session (for example, “Which step were you least sure about?”); and motivational messages were short, non-evaluative acknowledgements of persistence triggered by session length rather than by performance.

*Safeguards, fidelity and control condition*. Each week the instructors reviewed a random 5% of transcripts for factual errors and inappropriate content, and students could flag any response; 23 flagged responses (0.3% of all sessions) were reviewed during the semester, 9 of which contained factual inaccuracies that were corrected by adding material to the retrieval index. The model version and system prompt were frozen after a one-week pilot preceding the semester and were not changed during the study. Control sections used the standard teaching-assistant-moderated discussion forum in the same learning management system, with the same office hours as the intervention sections, and did not receive EduBot accounts. Because the two conditions differed on several dimensions at once (24/7 availability, Socratic questioning, personalisation, automated feedback and multimodality), the study evaluates an integrated AI-supported learning package rather than the isolated effect of Socratic questioning or of any single design component. We could not prevent students in either condition from using publicly available chatbots outside the course (see Limitations). Implementation fidelity was monitored through the platform logs (Table 2) and through weekly check-ins with the instructors, who confirmed each week that the control sections had no EduBot access.

**Table 2:** Per-student EduBot interaction metrics over the 84-day intervention period (intervention group, *n* = 278).

Metric	Mean	SD	Median	Min	Max	Skewness	Kurtosis
Total sessions	31.42	22.67	28.00	0	124	1.12	1.48
Average session duration (min)	8.74	4.92	7.82	0	28.45	0.87	0.64
Concept clarification requests	16.43	12.81	14.00	0	68	1.08	1.23
Socratic dialogue completions	12.16	9.74	10.00	0	52	1.21	1.56
Metacognitive prompt responses	2.83	2.91	2.00	0	16	1.45	2.31
Days active (out of 84 days)	28.64	16.23	26.00	0	72	0.52	−0.41

Each metric is the total per student (for average session duration, the student’s mean) over the 84 days of EduBot access (weeks 2–13); the columns summarise the distribution of these per-student values across the 278 intervention students. Days Active is the number of distinct days on which the student opened at least one session. Eighteen students (6.5%) recorded zero on all metrics.

### 2.3 Data collection instruments

*Student engagement*. We measured engagement at each wave with a 21-item scale adapted from Reeve and Tseng’s (2011) four-dimensional measure of engagement during learning activities, which is, to our knowledge, the most widely used instrument that includes an agentic dimension alongside the behavioural, emotional and cognitive dimensions (Reeve, 2013). The scale comprises behavioural (5 items, e.g., “I listen carefully in this course”), emotional (4 items, e.g., “I enjoy learning new things in this course”), cognitive (7 items, e.g., “When I study for this course, I try to connect what I am learning with my own experiences”) and agentic engagement (5 items, e.g., “During class I ask questions to help me learn”); one of the eight original cognitive items was removed after the expert review described below because two items were judged redundant in Chinese. Items were rated from 1 (strongly disagree) to 7 (strongly agree), and subscale scores are item means. Freda et al. (2023) informed the wording of items that refer to university rather than school contexts.

*Validity procedures*. Two bilingual researchers in educational psychology produced the Chinese version by translation and back-translation (Brislin, 1970); discrepancies were resolved by discussion with a third researcher. A panel of three faculty members in higher-education research rated the relevance and clarity of each item, and the scale was piloted with 42 students from a non-participating section, after which four items were reworded. We examined construct validity with confirmatory factor analysis (CFA) at each wave and tested measurement invariance across conditions and across waves in the sequence configural, metric and scalar (Putnick and Bornstein, 2016), using ΔCFI ≤ 0.010 and ΔRMSEA ≤ 0.015 as criteria for invariance (Chen, 2007). Internal consistency (Cronbach’s α) at T1 was 0.89 (behavioural), 0.86 (cognitive), 0.91 (emotional) and 0.84 (agentic), with similar values at T2 and T3 (Table 3).

**Table 3:** Confirmatory factor analyses and measurement invariance of the 21-item engagement scale (complete-case sample, *n* = 555).

Model	χ^2^ (df)	CFI	TLI	RMSEA	SRMR	ΔCFI	ΔRMSEA	Cronbach’s α (beh/cog/emo/agn)
Four-factor CFA, T1	277.76 (183)	0.984	0.982	0.031	0.036	—	—	0.89/0.87/0.90/0.85
Four-factor CFA, T2	232.73 (183)	0.992	0.990	0.022	0.030	—	—	0.89/0.87/0.91/0.86
Four-factor CFA, T3	261.55 (183)	0.987	0.985	0.028	0.033	—	—	0.89/0.87/0.91/0.85
Across conditions, T1: configural	472.47 (366)	0.982	0.980	0.032	0.043	—	—	—
Across conditions, T1: metric	490.37 (383)	0.982	0.980	0.032	0.046	+0.000	+0.000	—
Across conditions, T1: scalar	504.80 (400)	0.982	0.982	0.031	0.046	+0.000	−0.001	—
Across conditions, T3: configural	475.53 (366)	0.981	0.978	0.033	0.043	—	—	—
Across conditions, T3: metric	488.75 (383)	0.981	0.980	0.032	0.046	+0.000	−0.001	—
Across conditions, T3: scalar	516.48 (400)	0.979	0.978	0.032	0.046	−0.002	+0.000	—
Across waves, T1–T3: configural	772.04 (549)	0.988	0.986	0.027	0.033	—	—	—
Across waves, T1–T3: metric	804.09 (583)	0.988	0.987	0.026	0.036	+0.000	−0.001	—
Across waves, T1–T3: scalar	837.41 (617)	0.988	0.987	0.025	0.036	+0.000	−0.001	—

Maximum-likelihood estimation on item covariances and means; four correlated factors (behavioural 5 items, cognitive 7, emotional 4, agentic 5). Standardised loadings ranged from 0.650 to 0.870, and factor correlations from 0.420 to 0.550. Invariance across waves treats the three occasions as groups. Invariance criteria: ΔCFI ≤ 0.010 and ΔRMSEA ≤ 0.015 (Chen, 2007); all steps met both criteria, supporting scalar invariance across conditions and across time.

*Administration and inclusion criteria*. Students completed the questionnaires online, in Chinese, through the learning management system’s survey tool; each wave remained open for seven days with reminders on days 3 and 6; completion took 10–12 min and occurred outside class. Responses were linked to platform logs through a coded student identifier held by the research team and were not seen by instructors, and participation had no bearing on grades. Two attention-check items (e.g., “Please select ‘agree’ for this item”) were embedded in each wave. Questionnaires were included in the analysis if the student was enrolled in one of the eight sections, was 18 years or older, had consented and had completed all three waves; questionnaires were excluded if either attention check was failed or the completion time was under 3 min.

*Log data and qualitative data*. To supplement self-report, we aggregated per student, over the 84-day intervention period, the EduBot platform logs of total chat sessions, session duration, Socratic dialogue completions, concept clarification requests, metacognitive prompt responses and days active. After the intervention we conducted 45-min semi-structured interviews with 30 intervention students (7–8 per university, purposively sampled to vary in year of study and EduBot use), alongside three focus groups with 12 instructors and teaching assistants, to explore how participants experienced the tool and any changes in classroom climate.

### 2.4 Data analysis plan

We analysed the quantitative data in R 4.4 and IBM SPSS 29. Because students were nested within eight course sections, the primary analysis of the Group × Time effect used linear mixed-effects models with random intercepts for students and for sections, estimated by restricted maximum likelihood, with time (T1, T2, T3) as a categorical within-person factor and the Group × Time terms as the parameters of interest; a model with random time slopes at the section level was compared by likelihood-ratio test. Mixed-design ANOVAs (Group × Time), which assume independent observations, are retained for comparability with earlier work and are reported alongside the mixed-effects models. Effect sizes are reported at two levels, partial η^2^ for the Group × Time interaction, which describes the longitudinal effect, and Cohen’s d with 95% confidence intervals for the between-group difference at T3, which describes the standardised post-test difference; these answer different questions and are labelled accordingly. Because four related outcomes were tested, *p*-values for the four Group × Time contrasts at T3 were adjusted with the Holm procedure (Holm, 1979). We examined dose–response within the intervention group with hierarchical regressions predicting T3 engagement from EduBot sessions (per 10 sessions) after entering baseline engagement and covariates. We examined moderation by year of study (first year versus later years) and by cumulative GPA (continuous, centred) in models predicting T3 engagement from baseline engagement, covariates, university fixed effects, condition, the moderator and the Condition × Moderator product, with heteroskedasticity-robust standard errors; these analyses were not pre-registered and are reported as exploratory, with Holm adjustment across the eight interaction tests. Sensitivity analyses compared three analytical populations defined before the outcome analyses: the complete-case sample (n = 555); the intent-to-treat population (all 583 consenting students, analysed with the mixed-effects model using all available waves under a missing-at-random assumption); and a per-protocol sample restricted to intervention students who logged at least 12 EduBot sessions (an average of one per intervention week) together with all control students. Students who never used EduBot remained in the intervention group in the complete-case and intent-to-treat analyses. We analysed the qualitative transcripts with reflexive thematic analysis in NVivo (Dhakal, 2022), combining inductive coding with deductive codes derived from the four engagement dimensions. In the integration stage the qualitative themes were used to generate hypotheses about the experiences through which the intervention might relate to engagement (Noyes et al., 2019); the themes were not measured quantitatively and no mediation analysis was conducted.

## 3 Results

### 3.1 Preliminary analyses and AI usage patterns

The final analysis included 555 undergraduate students from four comprehensive Chinese universities. Baseline analyses confirmed no statistically significant differences between the intervention and control groups across all demographic and engagement variables (see Table 4). Because each university contributed a single course, university and course are reported as one characteristic in Table 4.

**Table 4:** Demographic and baseline characteristics of participants by group.

Characteristic	Intervention group (*n* = 278)	Control group (*n* = 277)	Test statistic	*p*-value
Age (years), Mean ± SD	19.82 ± 1.45	19.76 ± 1.51	*t*(553) = 0.49	0.621
Gender, *n* (%)			χ^2^(2) = 1.75	0.417
Female	158 (56.8%)	148 (53.4%)		
Male	115 (41.4%)	123 (44.4%)		
Non-binary/Other	5 (1.8%)	6 (2.2%)		
Year of Study, *n* (%)			χ^2^(3) = 1.96	0.583
First-year	112 (40.3%)	118 (42.6%)		
Second-year	98 (35.3%)	89 (32.1%)		
Third year	51 (18.3%)	56 (20.2%)		
Fourth year+	17 (6.1%)	14 (5.1%)		
Cumulative GPA, Mean ± SD	3.24 ± 0.58	3.29 ± 0.54	*t*(553) = −1.09	0.274
University (course), *n* (%)			χ^2^(3) = 0.67	0.880
Zhejiang University (Psychology)	72 (25.9%)	70 (25.3%)		
Nanjing University (Computer Science)	68 (24.5%)	71 (25.6%)		
Wuhan University (Business)	71 (25.5%)	73 (26.4%)		
Fudan University (Biology)	67 (24.1%)	63 (22.7%)		
English Proficiency (CET-4 score), Mean ± SD	512.45 ± 42.38	509.87 ± 44.21	*t*(553) = 0.70	0.483
Prior AI Tool Experience, *n* (%)			χ^2^(1) = 0.41	0.523
Yes	124 (44.6%)	116 (41.9%)		
No	154 (55.4%)	161 (58.1%)		
Baseline engagement scores, Mean ± SD				
Behavioural engagement	4.82 ± 1.03	4.79 ± 1.06	*t*(553) = 0.31	0.756
Cognitive engagement	4.91 ± 0.97	4.88 ± 0.99	*t*(553) = 0.38	0.704
Emotional engagement	5.04 ± 1.12	5.01 ± 1.09	*t*(553) = 0.29	0.772
Agentic engagement	4.45 ± 1.18	4.41 ± 1.15	*t*(553) = 0.42	0.675

SD = Standard Deviation; engagement subscales are item means on a 7-point scale (1–7); each university contributed one course, so university and course are the same characteristic; CET-4 = College English Test Band 4 (national standardised English proficiency examination in China).

Baseline engagement was moderate, with the lowest scores on the agentic subscale in both conditions. Confirmatory factor analyses supported the four-factor structure at every wave (CFI ≥ 0.984, RMSEA ≤ 0.031, SRMR ≤ 0.036), and metric and scalar invariance held across conditions and across waves (all ΔCFI ≤ 0.002 and ΔRMSEA ≤ 0.001; Table 3), indicating that the four dimensions were measured equivalently across groups and time. During the 12-week intervention, log data showed that most students used EduBot, mainly for concept clarification and Socratic tutoring, but use was positively skewed: a highly active subset accounted for a disproportionate share of interactions, and 18 students (6.5%) logged no session (Table 2; Figure 1). Table 2 reports per-student totals over the 84-day intervention period, summarised across the 278 intervention students.

**Figure 1:**
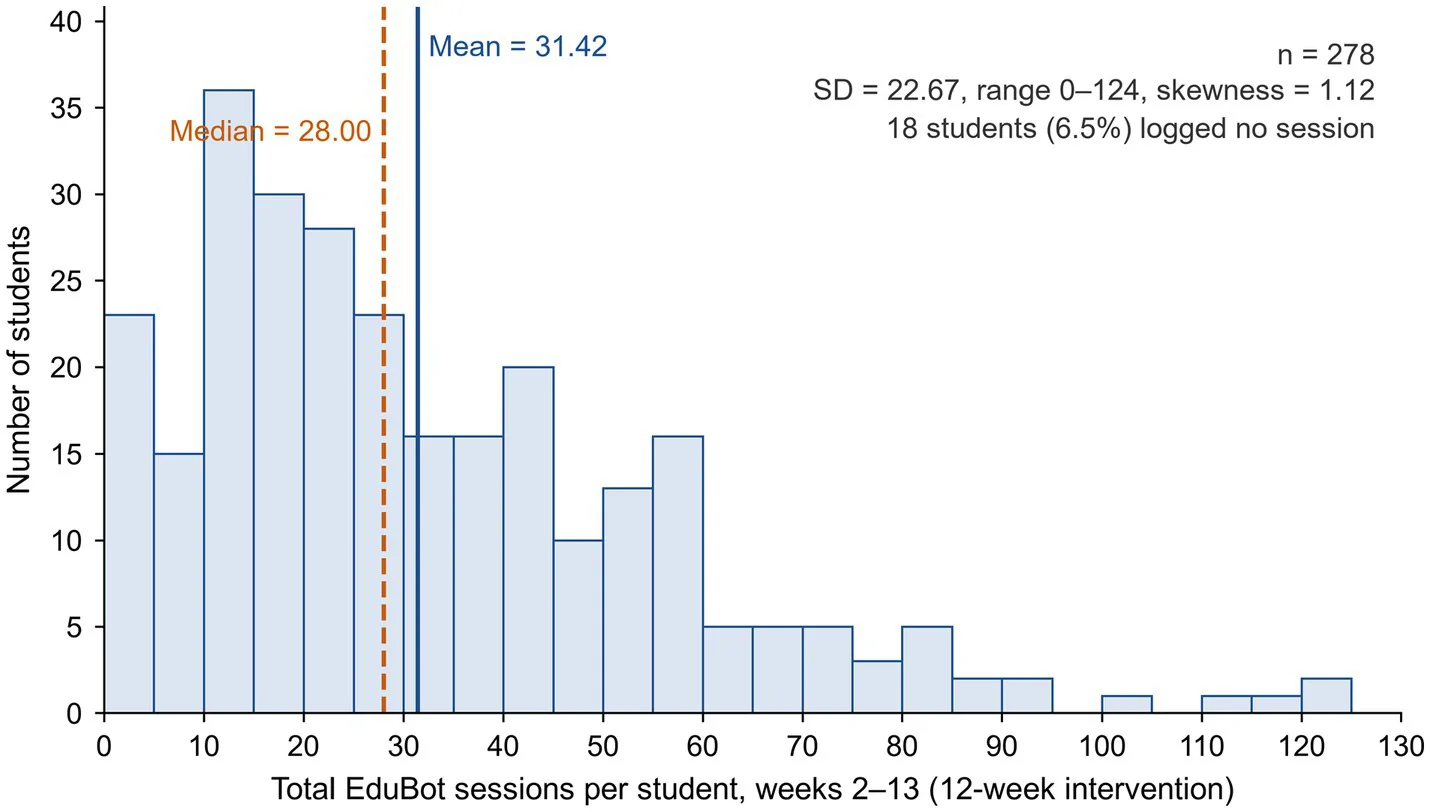
Distribution of total EduBot sessions per student over the 12-week intervention period (intervention group, *n* = 278). Vertical lines mark the mean (31.42) and the median (28.00); 18 students (6.5%) logged no session.

### 3.2 Quantitative effects of AI on student engagement

Mixed-design ANOVAs and linear mixed-effects models converged. Group × Time interactions were present for all four dimensions, with the intervention sections increasing from T1 to T3 while the control sections remained stable or declined slightly (Figure 2, Tables 1, 5, 6). In the mixed-effects models, which account for the nesting of students within sections, the Group × T3 contrast was 0.67 scale points for behavioural engagement (95% CI [0.51, 0.84]), 0.48 [0.33, 0.64] for cognitive, 0.34 [0.18, 0.49] for emotional and 0.55 [0.37, 0.72] for agentic engagement, all *p* < 0.001 after Holm adjustment (Table 1). Section-level ICCs were small (0.019–0.038), and the variance of section-level time slopes was estimated at zero in every model (likelihood-ratio χ^2^(2) = 0.00), so allowing random slopes did not change the estimates. The interaction effect sizes (partial η^2^, Table 6) were largest for agentic (0.054) and behavioural (0.043) engagement, followed by cognitive (0.032) and emotional engagement (0.012). The standardised post-test differences (Cohen’s d at T3, Table 6) were 0.71 [0.54, 0.88] for behavioural, 0.55 [0.38, 0.72] for cognitive, 0.57 [0.40, 0.74] for agentic and 0.34 [0.18, 0.51] for emotional engagement. Because the last measurement was taken in the week after EduBot access ended, these results describe within-semester trajectories and not effects sustained beyond the intervention.

**Figure 2:**
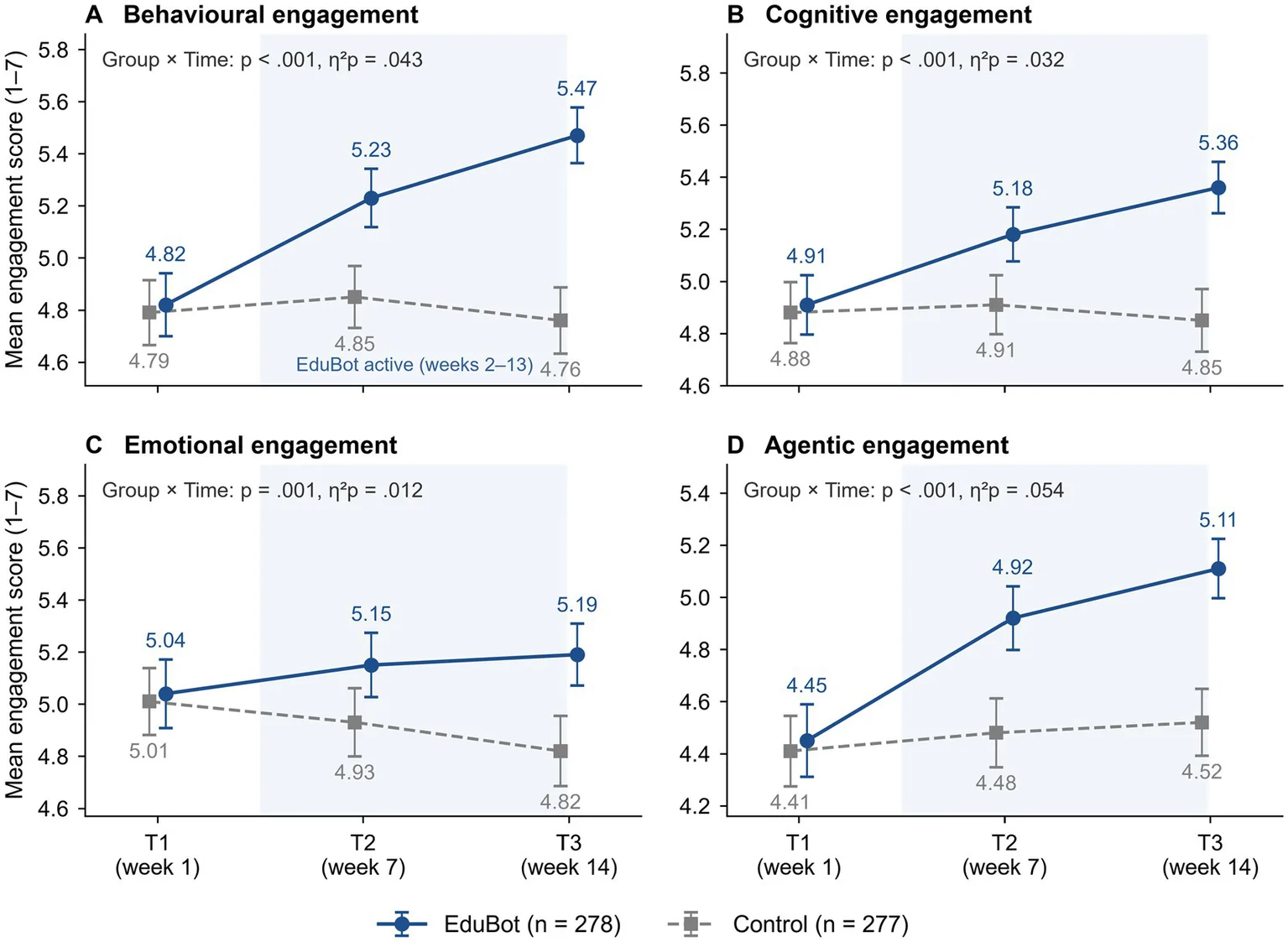
Longitudinal Changes in Student Engagement Dimensions by Group and Time. Points are means of the four engagement subscales (1–7) at T1 (week 1), T2 (week 7) and T3 (week 14); error bars are 95% confidence intervals; the shaded band marks the 12 weeks during which EduBot was available to the intervention sections. Group × Time statistics are from Table 6. **(A)** Behavioural engagement; **(B)** cognitive engagement; **(C)** emotional engagement; **(D)** agentic engagement.

**Table 5:** Descriptive statistics for engagement subscales by group and time point.

Engagement dimension	Group	Pre-intervention mean (SD)	Mid-intervention mean (SD)	Post-intervention mean (SD)
Behavioural engagement	Intervention	4.82 (1.03)	5.23 (0.95)	5.47 (0.91)
	Control	4.79 (1.06)	4.85 (1.01)	4.76 (1.08)
Cognitive engagement	Intervention	4.91 (0.97)	5.18 (0.88)	5.36 (0.84)
	Control	4.88 (0.99)	4.91 (0.96)	4.85 (1.02)
Emotional engagement	Intervention	5.04 (1.12)	5.15 (1.05)	5.19 (1.01)
	Control	5.01 (1.09)	4.93 (1.11)	4.82 (1.14)
Agentic engagement	Intervention	4.45 (1.18)	4.92 (1.04)	5.11 (0.97)
	Control	4.41 (1.15)	4.48 (1.12)	4.52 (1.09)

All scores are on a 7-point Likert scale. SD = Standard Deviation.

**Table 6:** Summary of mixed ANOVA results for engagement outcomes, with standardised between-group differences at T3.

Engagement dimension	Effect	df	F	*p*	Partial η^2^	Cohen’s d at T3 [95% CI]
Behavioural engagement	Time	2, 1,106	8.92	< 0.001	0.016	—
	Group	1, 553	12.34	< 0.001	0.022	—
	Group × Time	2, 1,106	24.83	< 0.001	0.043	0.71 [0.54, 0.88]
Cognitive engagement	Time	2, 1,106	6.78	0.001	0.012	—
	Group	1, 553	9.87	0.002	0.018	—
	Group × Time	2, 1,106	18.37	< 0.001	0.032	0.55 [0.38, 0.72]
Emotional engagement	Time	2, 1,106	3.21	0.041	0.006	—
	Group	1, 553	5.64	0.018	0.010	—
	Group × Time	2, 1,106	6.92	0.001	0.012	0.34 [0.18, 0.51]
Agentic engagement	Time	2, 1,106	14.56	< 0.001	0.026	—
	Group	1, 553	18.23	< 0.001	0.032	—
	Group × Time	2, 1,106	31.45	< 0.001	0.054	0.57 [0.40, 0.74]

All *p*-values are two-tailed. Greenhouse–Geisser corrections have been used in case of violation of assumption of sphericity. Partial η^2^ describes the Group × Time interaction (the longitudinal effect); Cohen’s d is the standardised difference between groups at T3 (pooled SD, computed from Table 5) and does not describe the interaction. Holm-adjusted *p*-values for the four Group × Time interactions were all ≤ 0.001. The ANOVA assumes independent observations; mixed-effects models that account for course sections are reported in Table 1.

The largest relative change occurred in agentic engagement, which started from the lowest baseline in both groups. Emotional engagement showed the smallest difference, suggesting that affective connection to the course was less responsive to the tool than the behavioural, cognitive and agentic dimensions. Within the intervention group, EduBot use was related to engagement in a dose–response pattern (Tables 7, 8). After adjusting for baseline engagement, age, gender, year of study, GPA and prior AI experience, each additional 10 sessions was associated with higher T3 behavioural (b = 0.16, 95% CI [0.12, 0.20], β = 0.370), cognitive (b = 0.12, 95% CI [0.09, 0.16], β = 0.310), agentic (b = 0.14, 95% CI [0.10, 0.18], β = 0.280) and, to a smaller degree, emotional engagement (b = 0.06, 95% CI [0.02, 0.10], β = 0.130), with ΔR^2^ of 0.016–0.136; Socratic dialogue completions showed the same pattern. Table 9 reports the group effect in the full sample and does not include usage variables, which are defined only for the intervention group. The dose–response associations are correlational within a self-selected usage distribution and do not establish that additional use caused additional engagement.

**Table 7:** Pearson correlation matrix: EduBot interaction metrics and post-intervention engagement scores (intervention group only, *n* = 278).

Variable	1	2	3	4	5	6	7	8	9	10
1. Total sessions	—									
2. Avg session duration	0.34	—								
3. Concept clarification	0.89	0.28	—							
4. Socratic dialogue	0.82	0.41	0.62	—						
5. Metacognitive prompts	0.58	0.19	0.48	0.51	—					
6. Days active	0.91	0.37	0.82	0.78	0.54	—				
7. Behavioural engagement	0.42	0.21	0.38	0.35	0.22	0.44	—			
8. Cognitive engagement	0.36	0.25	0.32	0.38	0.28	0.39	0.52	—		
9. Emotional engagement	0.15	0.08	0.13	0.17	0.11	0.19	0.38	0.34	—	
10. Agentic engagement	0.48	0.23	0.51	0.42	0.31	0.52	0.47	0.41	0.22	—

*n* = 278, |*r*| ≥ 0.12 is significant at *p* < 0.05, |*r*| ≥ 0.16 at *p* < 0.01 and |*r*| ≥ 0.20 at *p* < 0.001 (two-tailed).

**Table 8:** Dose–response associations between EduBot use and T3 engagement within the intervention group (*n* = 278).

Outcome (T3)	Usage predictor (Block 2)	b (SE)	95% CI	β	ΔR^2^ (Block 2)	Total R^2^	*p*
Behavioural engagement	Total sessions (per 10)	0.16 (0.02)	[0.12, 0.20]	0.370	0.136	0.409	< 0.001
	Socratic dialogue completions (per 10)	0.35 (0.05)	[0.25, 0.44]	0.350	0.121	0.394	< 0.001
Cognitive engagement	Total sessions (per 10)	0.12 (0.02)	[0.09, 0.16]	0.310	0.091	0.371	< 0.001
	Socratic dialogue completions (per 10)	0.26 (0.05)	[0.17, 0.35]	0.280	0.074	0.354	< 0.001
Emotional engagement	Total sessions (per 10)	0.06 (0.02)	[0.02, 0.10]	0.130	0.016	0.413	0.007
	Socratic dialogue completions (per 10)	0.11 (0.05)	[0.01, 0.21]	0.110	0.011	0.408	0.027
Agentic engagement	Total sessions (per 10)	0.14 (0.02)	[0.10, 0.18]	0.280	0.076	0.475	< 0.001
	Socratic dialogue completions (per 10)	0.27 (0.05)	[0.17, 0.38]	0.230	0.053	0.452	< 0.001

Hierarchical ordinary least squares regressions. Block 1: baseline score of the outcome, age, gender, year of study, GPA and prior AI experience; Block 2: the usage predictor. Because the usage metrics are strongly intercorrelated (Table 7), each is entered in a separate model. *p* for total sessions is Holm-adjusted across the four outcomes; p for Socratic completions is unadjusted. Associations are correlational within a self-selected distribution of use.

**Table 9:** Hierarchical multiple regression analysis predicting post-intervention engagement (full sample, *N* = 555) (usage variables are not included because they are defined only for the intervention group; see Table 8).

Predictor	Behavioural engagement β	Cognitive engagement β	Emotional engagement β	Agentic engagement β
Block 1: Covariates				
Age	−0.03	−0.02	−0.04	−0.01
Gender (Female = 1)	0.05	0.03	0.08	0.04
Year of study	−0.06	−0.04	−0.07	−0.05
Cumulative GPA	0.12	0.14	0.09	0.08
Prior AI experience (Yes = 1)	0.07	0.05	0.04	0.09
Baseline engagement score	0.42	0.38	0.46	0.35
Block 2: Group				
Group (Intervention = 1)	0.26	0.22	0.15	0.32
ΔR^2^ for Block 2	0.06	0.04	0.02	0.09
Model summary (Block 2)				
Total R^2^	0.38	0.32	0.29	0.41
Adjusted R^2^	0.36	0.30	0.27	0.39
*F* (8, 546)	41.83	32.15	27.89	47.42

β = standardised regression coefficient.

Exploratory moderation analyses (Table 10) found two of eight Condition × Moderator interactions to be significant after Holm adjustment. The association between condition and T3 agentic engagement was larger among first-year students (b = 0.44, 95% CI [0.12, 0.76], adjusted *p* = 0.046); the simple effect of condition was 0.82 (SE 0.12) scale points for first-year and 0.38 (SE 0.10) for later-year students. The association with T3 cognitive engagement decreased with GPA (b = −0.47 per GPA point, 95% CI [−0.73, −0.21], adjusted *p* = 0.003); the simple effect was 0.75 (SE 0.10) at one SD below the GPA mean and 0.24 (SE 0.10) at one SD above. No other interaction reached significance (all adjusted *p* ≥ 0.431). Sensitivity analyses (Table 11) gave Group × T3 estimates within 0.01 scale points of the complete-case estimates in the intent-to-treat population (n = 583) and 0.03–0.09 points larger in the per-protocol sample, consistent with the dose–response pattern.

**Table 10:** Exploratory moderation of the condition effect on T3 engagement by year of study and cumulative GPA (complete-case sample, *n* = 555).

Outcome (T3)	Interaction term	b (SE)	95% CI	*p*	Holm-adjusted p	Simple effect of condition: first-year/GPA −1 SD, b (SE)	Simple effect of condition: later years/GPA +1 SD, b (SE)
Behavioural engagement	Condition × First-year	−0.06 (0.16)	[−0.37, 0.25]	0.720	1.000	0.65 (0.13)	0.71 (0.10)
	Condition × GPA (per point)	−0.04 (0.14)	[−0.31, 0.23]	0.748	1.000	0.71 (0.11)	0.66 (0.11)
Cognitive engagement	Condition × First-year	0.26 (0.14)	[−0.02, 0.54]	0.072	0.431	0.65 (0.11)	0.39 (0.09)
	Condition × GPA (per point)	−0.47 (0.13)	[−0.73, −0.21]	< 0.001	0.003	0.75 (0.10)	0.24 (0.10)
Emotional engagement	Condition × First-year	0.05 (0.15)	[−0.24, 0.34]	0.746	1.000	0.38 (0.11)	0.33 (0.10)
	Condition × GPA (per point)	−0.11 (0.14)	[−0.38, 0.17]	0.436	1.000	0.41 (0.11)	0.29 (0.10)
Agentic engagement	Condition × First-year	0.44 (0.16)	[0.12, 0.76]	0.007	0.046	0.82 (0.12)	0.38 (0.10)
	Condition × GPA (per point)	−0.09 (0.15)	[−0.39, 0.21]	0.553	1.000	0.62 (0.11)	0.52 (0.12)

Ordinary least squares with heteroskedasticity-consistent (HC3) standard errors; each model adjusts for the baseline score of the outcome, age, gender, prior AI experience, university fixed effects and, for the year models, GPA (for the GPA models, year of study). GPA is centred (SD = 0.54); simple effects for GPA are evaluated at one SD below and above the mean. Holm adjustment is across the eight interaction tests. These analyses were not pre-registered and are exploratory.

**Table 11:** Sensitivity analyses: Group × T3 estimates from the mixed-effects model in three analytical populations.

Engagement dimension	Complete case (*n* = 555), b [95% CI]	Intent-to-treat (*n* = 583), b [95% CI]	Per-protocol (*n* = 500), b [95% CI]
Behavioural engagement	0.67 [0.51, 0.84]	0.66 [0.50, 0.83]	0.76 [0.59, 0.94]
Cognitive engagement	0.48 [0.33, 0.64]	0.48 [0.32, 0.63]	0.56 [0.40, 0.72]
Emotional engagement	0.34 [0.18, 0.49]	0.35 [0.20, 0.51]	0.36 [0.20, 0.53]
Agentic engagement	0.55 [0.37, 0.72]	0.54 [0.36, 0.71]	0.63 [0.45, 0.82]

Complete case: students with all three waves (primary analysis, Table 1). Intent-to-treat: all 583 consenting students analysed as allocated, using every available wave under a missing-at-random assumption (28 students missing T3, of whom 14 also missed T2). Per-protocol: the 223 intervention students (80.2%) who logged at least 12 sessions (one per intervention week on average) plus all 277 control students; the 55 intervention students below the threshold, including the 18 who never used EduBot, were excluded. The threshold was fixed before the outcome analyses. All *p* < 0.001.

### 3.3 Qualitative mechanisms and mixed-methods integration

Reflexive thematic analysis of interview and focus group transcripts identified three themes that participants used to explain their experience with EduBot; we treat these as hypothesised mechanisms rather than as tested ones (see Table 12 for representative quotations):

**Table 12:** Representative quotations from qualitative data by theme.

Theme	Participant	Quotation
Theme 1: The Judgment-Free Zone and Face-Saving Dynamics	Student 7 (Female, Year 2, Zhejiang University)	“In our Chinese classrooms, we do not like to interrupt the teacher. It feels disrespectful. But with EduBot, I could ask the same question many times without losing face. It was a sort of a one-on-one tutor, one that never loses his temper.”
	Student 14 (Male, Year 1, Fudan University)	“Sometimes self-directed learning is superficial, blind, and inefficient. EduBot made me feel safe to be curious again.”
	Student 19 (Female, Year 3, Nanjing University)	“Sometimes I do not even know what I do not understand. EduBot let me explore my confusion without embarrassing myself in front of classmates.”
Theme 2: Scaffolded Persistence and Shifting Epistemological Beliefs	Student 22 (Male, Year 3, Nanjing University)	“I initially wanted it to simply provide me with the right answer fast. That is what we are used to. Then after some time I suddenly understood that I was even following the logic. It was similar to being on a mountain climbing step by step with a tutor.”
	Student 29 (Female, Year 2, Wuhan University)	“I would simply repeat what is in the back of the textbook. EduBot would not allow me to do that. It made me think, and at first doing so was a pain, but useful in the long run.”
	Student 4 (Male, Year 1, Zhejiang University)	“The AI did not judge me for being slow. It just kept asking me questions until I figured it out myself. That’s very different from how we learned in high school.”
Theme 3: Temporal Flexibility and the Demands of Chinese Student Life	Instructor (Focus Group, Zhejiang University)	“My students are very busy. Many of them have part-time jobs in the evenings. The fact that they could get help from EduBot at 11 p.m. when I was sleeping was a game changer for their learning continuity.”
	Student 11 (Female, Year 4, Fudan University)	“I am preparing for the postgraduate entrance exam while taking this course. I do not have time to attend every office hour. EduBot was always there for me.”
	TA (Focus Group, Wuhan University)	“The volume of repetitive questions in office hours dropped dramatically. Students were coming to us with deeper questions because the AI had already handled the basics.”

(1) The judgment-free zone: students described EduBot as an interlocutor with whom they could ask, and repeat, questions without the social cost they associated with interrupting a lecturer or exposing confusion to classmates; (2) Scaffolded persistence: by withholding direct answers, the Socratic design was experienced as pushing against an answer-seeking habit that several students traced to examination-oriented secondary schooling, and as leading to deeper processing; and (3) Temporal flexibility: 24/7 availability fitted around part-time work, postgraduate-entrance-examination preparation and other demands on students’ time. Figure 3 summarises these themes as hypothesised pathways linking the delivered design features to the measured engagement outcomes. The pathways in Figure 3 were derived from the qualitative data and were not tested statistically; the themes were not measured quantitatively, and the figure should not be read as a mediation model.

**Figure 3:**
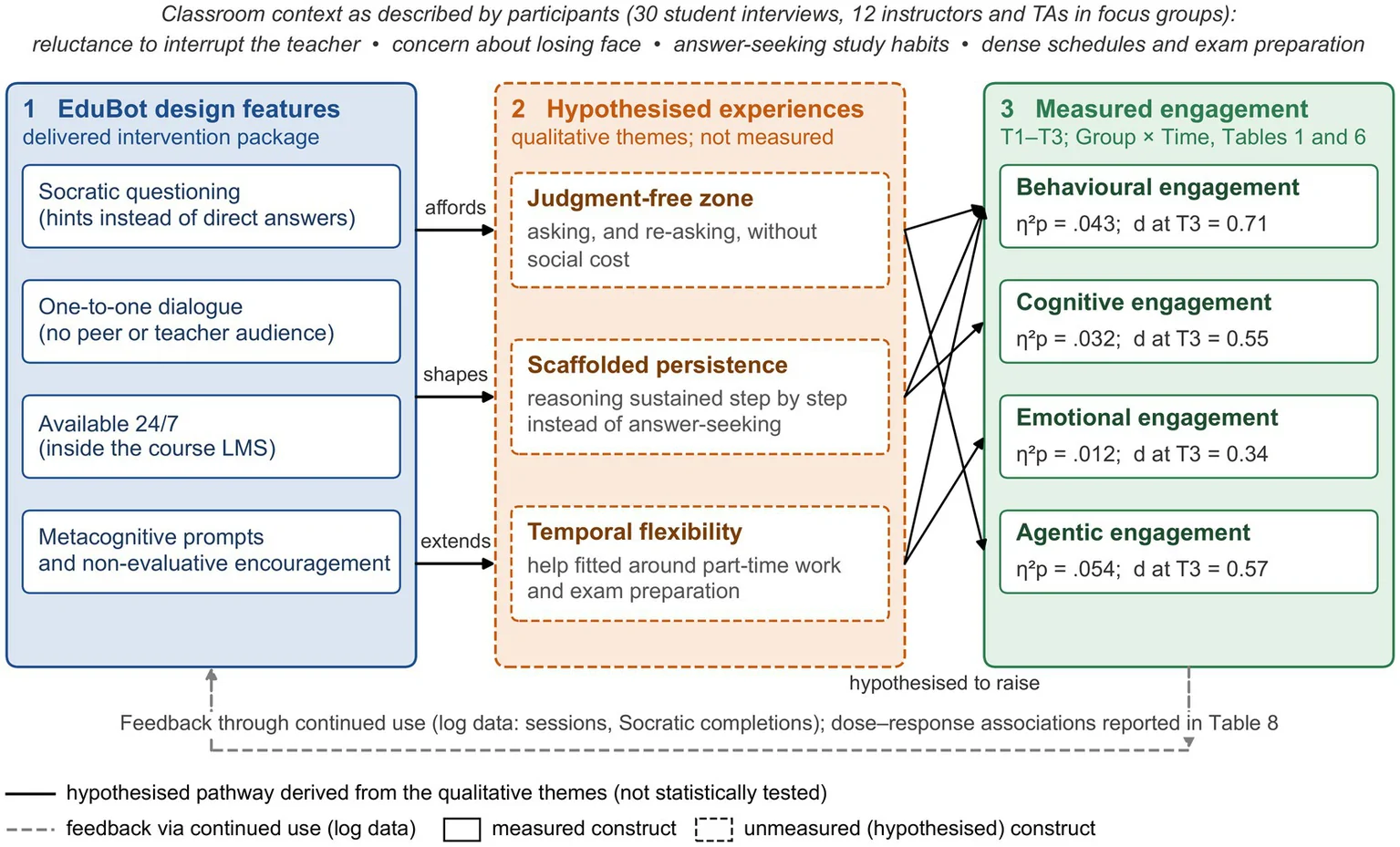
Hypothesised model of the relations between EduBot design features, the experiences described by participants and the measured engagement outcomes. Solid arrows are pathways proposed on the basis of the qualitative themes and were not statistically tested; the constructs in the middle column were not measured; grey dashed arrows show the feedback through continued use, for which dose–response associations are reported in Table 8. η^2^p values are the Group × Time interaction effect sizes from Table 6.

## 4 Discussion

The current research examined associations between AI-based conversational assistant use and student engagement in Chinese higher education. Using a mixed-method quasi-experimental design with 555 undergraduate students across four major universities, the findings provide evidence consistent with the view that thoughtfully designed AI tools are associated with meaningful engagement differences in behavioural, cognitive, and agentic dimensions, and a smaller but significant difference in emotional engagement. This discussion contextualises the main findings within the existing literature, examines three hypothesised mechanisms that emerged from the qualitative data, namely (i) the AI tool’s role as a face-saving, judgment-free interlocutor that lowers the social cost of asking questions within classroom hierarchies; (ii) its capacity to scaffold persistence and gradually shift the answer-seeking habits that several participants traced to examination-oriented secondary schooling toward more inquiry-oriented learning behaviours; and (iii) its temporal flexibility, which accommodates the dense academic schedules, postgraduate-entrance-exam preparation, and part-time work commitments characteristic of contemporary Chinese undergraduate life, and addresses the limitations of this study alongside implications for educational practice and policy in China and beyond.

### 4.1 Interpretation of engagement outcomes in the Chinese context

The largest interaction effect was observed for agentic engagement (partial η^2^ = 0.054; post-test d = 0.57, 95% CI [0.40, 0.74]), the dimension with the lowest baseline in both groups. Agentic engagement refers to students’ proactive contribution to instruction, for example by asking questions, expressing preferences and requesting clarification (Reeve and Tseng, 2011; Wang and Lee, 2021). The judgment-free-zone theme offers one plausible explanation. Interviewees described EduBot as an interaction partner that allowed them to ask questions without the embarrassment or perceived disrespect they associated with interrupting an instructor, which is consistent with studies linking face concerns and social anxiety to classroom silence in Chinese universities (Fang and Tang, 2021; Liu et al., 2026; Shi, 2026). On this reading, the tool provided a patient, private channel for questioning that some students had previously avoided (Meyer, 2023; Zhang, 2025), and it adds to the small set of pedagogical interventions reported to reduce reticence in Chinese university classrooms. Two qualifications apply. First, the themes were derived from 30 interviews and were not measured, so the pathway from the tool to agentic engagement through reduced face concern is a hypothesis for future testing rather than a demonstrated mechanism. Second, the accounts are situated in four courses at four research-intensive universities; classroom norms differ across institutions, disciplines and cohorts, and an account of “Chinese classroom culture” as a single obstacle would overstate what these data can show (Ryan and Louie, 2007).

The differences in cognitive (d = 0.55) and behavioural engagement (d = 0.71) are consistent with the intended function of Socratic dialogue. The scaffolded-persistence theme showed that students initially sought direct answers, a habit several attributed to examination-oriented secondary schooling (Belland et al., 2013; Liu and Lian, 2026), and that continued exposure to EduBot’s questioning was experienced as a gradual shift toward working through problems. This description is consistent with Vygotskian accounts of scaffolding within the zone of proximal development (Belland et al., 2013) and with the argument that AI tools designed to guide rather than to answer may support the critical-thinking goals of current Chinese higher-education reform (Bamasoud et al., 2025). The dose–response associations between Socratic dialogue completions and T3 cognitive engagement (Table 8) are in line with this interpretation, although they are correlational and could partly reflect that more engaged students chose to use the tool more.

The smaller difference in emotional engagement (d = 0.34) is theoretically interpretable. Emotional engagement involves belonging, interest and affective attachment to the learning community (Santos et al., 2023; Zhang, 2023), and such attachments are formed largely through relationships with teachers and peers rather than through a software interface (Wang et al., 2025). EduBot was associated with gains in cognitive and behavioural engagement but did not appear to substitute for the relational processes that underpin emotional investment. This pattern argues against accounts in which AI becomes a one-stop solution to disengagement and instead suggests a complementary model in which AI tools address specific cognitive and behavioural needs, leaving instructors more room for the relational and affective aspects of teaching (Sun et al., 2025).

### 4.2 Moderation by student characteristics: exploratory findings

The exploratory moderation analyses indicated that the association between condition and agentic engagement was larger among first-year students, and that the association with cognitive engagement was larger among students with lower prior GPA (Table 10). The first-year pattern is consistent with transition research, which characterises the first year of university as a period of adjustment in which engagement is vulnerable (Worsley et al., 2021), and with the move from the more structured, teacher-directed study of senior secondary school to the greater autonomy expected at university. One interpretation is that the tool offered a low-risk way to seek help and to practise self-regulated learning at a stage when students are least confident about revealing difficulties to peers or professors.

The larger cognitive association among students with lower prior GPA is of interest for equity. Academic competition is intense in many Chinese universities, and institutional support is not always sufficient for lower-achieving students (Li et al., 2025); a tool that offers additional scaffolding to those who need it most could play a compensatory role, in line with scholarship that sees AI as a possible means of narrowing educational disparities when it is deployed equitably (Nedungadi et al., 2024). Three cautions apply. The interactions were not pre-registered, only two of eight survived adjustment for multiple testing, and the confidence intervals are wide; the findings therefore warrant replication before they inform policy. In addition, the 6.5% of intervention students who never used EduBot may be a group for whom access, digital skills or motivation limited use, and future studies should examine the characteristics of non-users.

### 4.3 Limitations and future directions

Several limitations qualify the findings. First, the design was quasi-experimental: sections rather than students were assigned to conditions, only eight sections were involved, and each university contributed a single course, so university and discipline are perfectly confounded and institutional or disciplinary effects cannot be separated from the intervention. Although the conditions were balanced on all measured covariates at baseline, unmeasured section-level differences cannot be excluded, and the cluster-adjusted power calculation shows that the design was adequately powered only for medium-to-large effects. Cluster-randomised trials with more sections, and with courses replicated across institutions, would strengthen causal inference. Second, the intervention was an integrated package that differed from the control condition on several dimensions at once, so the effects cannot be attributed to Socratic questioning, availability, personalisation or any other single component; component-level designs are needed. Third, the last measurement was taken in the week after EduBot access ended within one 15-week semester, so the results describe within-semester trajectories, and whether the differences persist, fade or grow after the intervention is unknown. Fourth, engagement was self-reported; although log data provide an objective record of use for the intervention group and measurement invariance held across groups and waves, common-method variance may inflate associations, and future work should add academic performance, retention and classroom observation. Fifth, the three qualitative themes were not measured quantitatively, and no mediation analysis was possible; the pathways in Figure 3 are hypotheses. Sixth, we could not prevent students in either condition from using publicly available chatbots outside the course, which would, if anything, attenuate the observed differences. Finally, the cultural interpretations rest on 30 interviews in four courses and should be read as participants’ situated accounts rather than as characteristics of Chinese higher education in general.

### 4.4 Implications for practice and policy

The findings have several implications for educational practice and policy in Chinese higher education. To teachers and curriculum developers, the research highlights the need to implement AI tools as more overtly pedagogical scaffolds as opposed to placing them in the role of robots that provide answers. Priority should be given to Socratic dialogue capability, concept clarifying prompts and metacognitive questioning, compared to straightforward generation of the answer. To institutional leaders and policymakers, the exploratory finding of larger associations among lower-performing students suggests, pending replication, that AI learning-support systems may be worth prioritising in institutions and student groups with the greatest need (Oncioiu and Bularca, 2025). The judgment-free-zone theme also underlines the importance of culturally aware design of learning technology. Applications of AI developed in other educational settings may not transfer directly to Chinese classrooms without attention to local communication norms, power distance and face, which themselves vary across institutions and disciplines.

## 5 Conclusion

This study provides evidence that access to an AI conversational assistant with pedagogical scaffolding was associated with higher behavioural, cognitive, agentic and, to a smaller extent, emotional engagement among undergraduates in four Chinese universities over a semester, in analyses that account for the nesting of students within course sections. The clearest association was for agentic engagement, the dimension that participants described as most constrained by classroom norms, which suggests that a private, patient questioning channel may support student voice in settings where asking in class carries a social cost. Exploratory analyses indicated larger associations among first-year students and among students with lower prior achievement; these findings require replication before they can support equity claims. Qualitative accounts pointed to three hypothesised mechanisms, a judgment-free interaction space, scaffolded persistence and temporally flexible support, which remain to be tested with measured mediators. The modest association with emotional engagement is a reminder that such tools complement, rather than substitute for, the relational dimensions of teaching. As Chinese universities integrate artificial intelligence into teaching, these findings describe AI as a pedagogical supplement that may support engagement while leaving the human dimensions of education in place.

## Data Availability

The datasets presented in this article are not readily available because they contain student-level questionnaire responses, EduBot platform logs and interview transcripts that are protected by institutional data-privacy policies and the terms of participant consent. Requests to access the datasets should be directed to Jing Dong, jingd@yzu.edu.cn.
